# Supplementary material for: DHRS7 Integrates NADP+/NADPH Redox Sensing with Inflammatory Lipid Signalling via the Oxoeicosanoid Pathway
Source: bioRxiv. 2025 Feb 8:2025.02.05.636725. Preprint. [Version 1] doi: 10.1101/2025.02.05.636725 (PMC11839141; doi:10.1101/2025.02.05.636725)
Supplement: Supplement 1 [file NIHPP2025.02.05.636725v1-supplement-1.pdf]

bioRxiv preprint doi: https://doi.org/10.1101/2025.02.05.636725; this version posted February 8, 2025. The copyright holder for this preprint (which was not certified by peer review) is the author/funder, who has granted bioRxiv a license to display the preprint in perpetuity. It is made available under aCC-BY 4.0 International license.

Figure S1

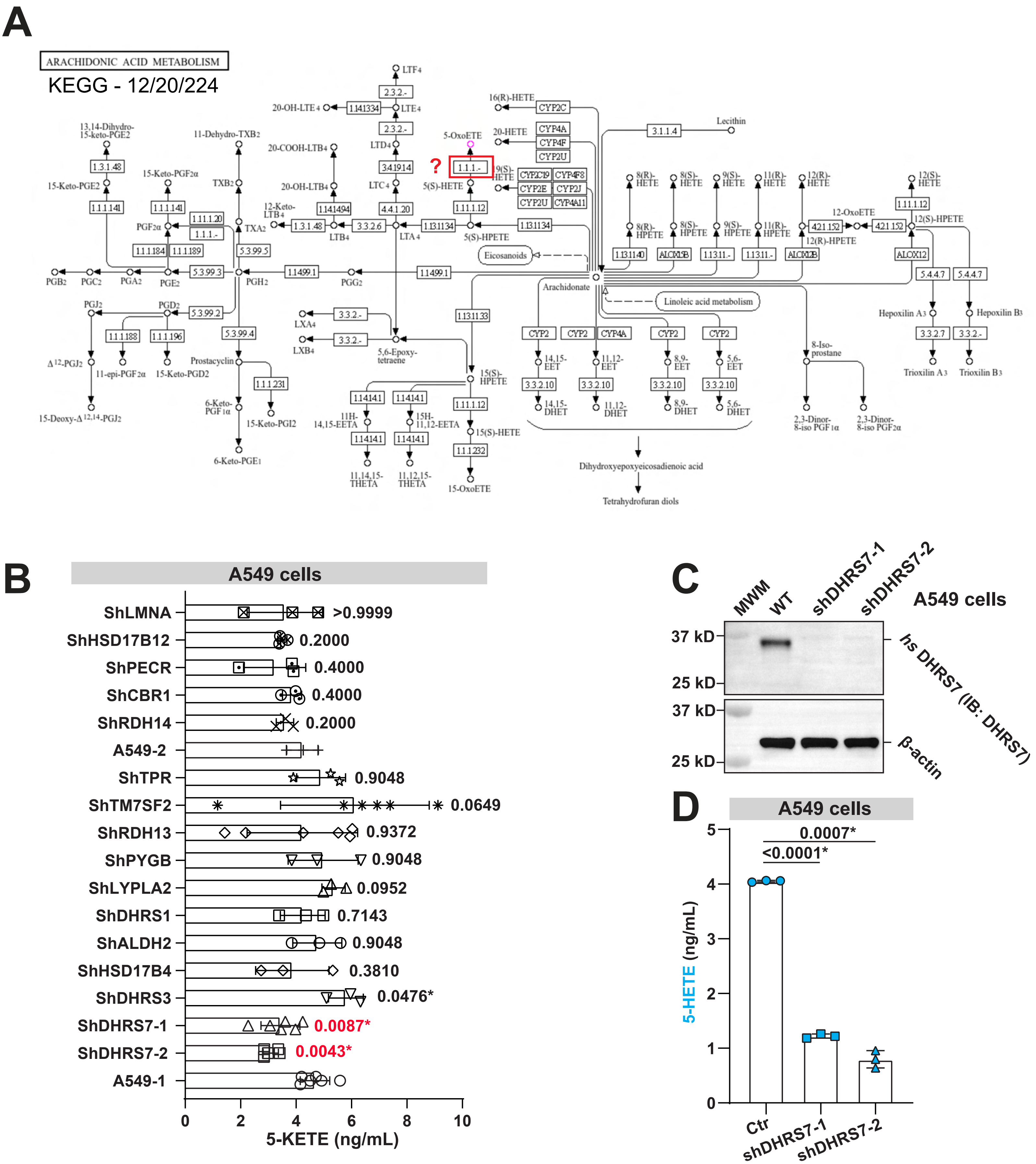

bioRxiv preprint doi: https://doi.org/10.1101/2025.02.05.636725; this version posted February 8, 2025. The copyright holder for this preprint (which was not certified by peer review) is the author/funder, who has granted bioRxiv a license to display the preprint in perpetuity. It is made available under aCC-BY 4.0 International license.

Figure S1. 5-HEDH candidate screen. Related to Figure 1.

(A) KEGG pathway scheme of arachidonic acid pathway showing the position of 5-HEDH (red). **(B)** Limited candidate screen for 5-HEDH using shRNA gene interference and LC-MS in A549 cells. Lipids extractions from shDHRS7-1, shDHRS7-2, shDHRS3, shHSD17B4, shALDH2, shDHRS1, shLYPLA2, shPYGB, shRDH13, shTM7SF2, shTPR were compared to A549-1, while shRDH14, shCBR1, shPECR, shHSD17B12, shLMNA were compared to A549-2. P values, unpaired, nonparametric, two tailed Mann-Whitney Test. **(C)** Western blot confirming DHRS7 knockdown by the two shRNAs (shDHRS7-1 and shDHRS7-2) used in the screen. β-actin was used as an internal control. MWM, molecular weight marker. **(D)** LC-MS assay showing 5-HETE production in DHRS7 knockdown A549 cells upon incubation with 1 μM 5-KETE. P values, unpaired, parametric, two tailed Welch's t test. Asteriks, highlight significant changes as compared to respective control ($p<0.05$).

Figure S2

bioRxiv preprint doi: https://doi.org/10.1101/2025.02.05.636725; this version posted February 8, 2025. The copyright holder for this preprint (which was not certified by peer review) is the author/funder, who has granted bioRxiv a license to display the preprint in perpetuity. It is made available under aCC-BY 4.0 International license.

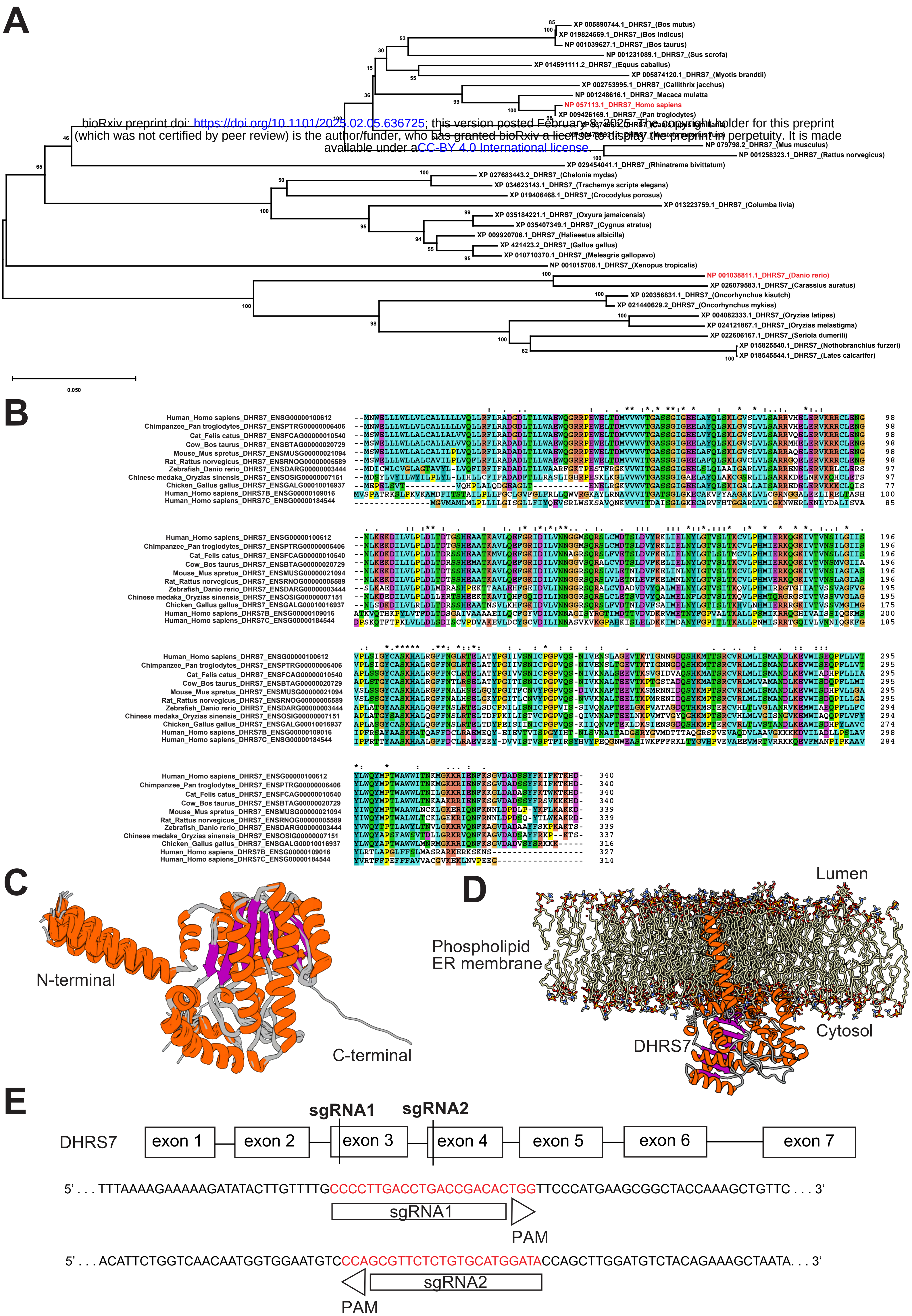

bioRxiv preprint doi: https://doi.org/10.1101/2025.02.05.636725; this version posted February 8, 2025. The copyright holder for this preprint (which was not certified by peer review) is the author/funder, who has granted bioRxiv a license to display the preprint in perpetuity. It is made available under aCC-BY 4.0 International license.

Figure S2. Phylogenetic analysis of DHRS7. Related to Figure 1.

(A) Phylogenetic tree analysis of DHRS7 including human (*homo sapiens*), zebrafish (*danio rerio*) and 32 other species. **(B)** Protein sequence alignment of human DHRS7, DHRS7B, DHRS7C, zebrafish DHRS7 and orthologs from 7 other species. Asterisks (*), fully conserved residues. Colons (:) strongly similar conserved residues. Periods (.), weakly similar conserved residues. **(C)** Superimposed AlphaFold2 structures of 34 DHRS7 orthologs (shown in S2A). The colour key corresponds to alpha-helices (orange), beta-sheets (purple) and loops or unstructured (grey). **(D)** Cartoon model depicting the predicted structure of DHRS7 (ID:AF-Q9Y394-F1-v4), anchored in simulated ER phospholipid membrane leaflets **(E)** Schematic representation of DHRS7 CRISPR design. Two sgRNAs were designed to target exon 3 and exon 4.

bioRxiv preprint doi: https://doi.org/10.1101/2025.02.05.636725; this version posted February 8, 2025. The copyright holder for this preprint (which was not certified by peer review) is the author/funder, who has granted bioRxiv a license to display the preprint in perpetuity. It is made available under aCC-BY 4.0 International license.

Figure S3

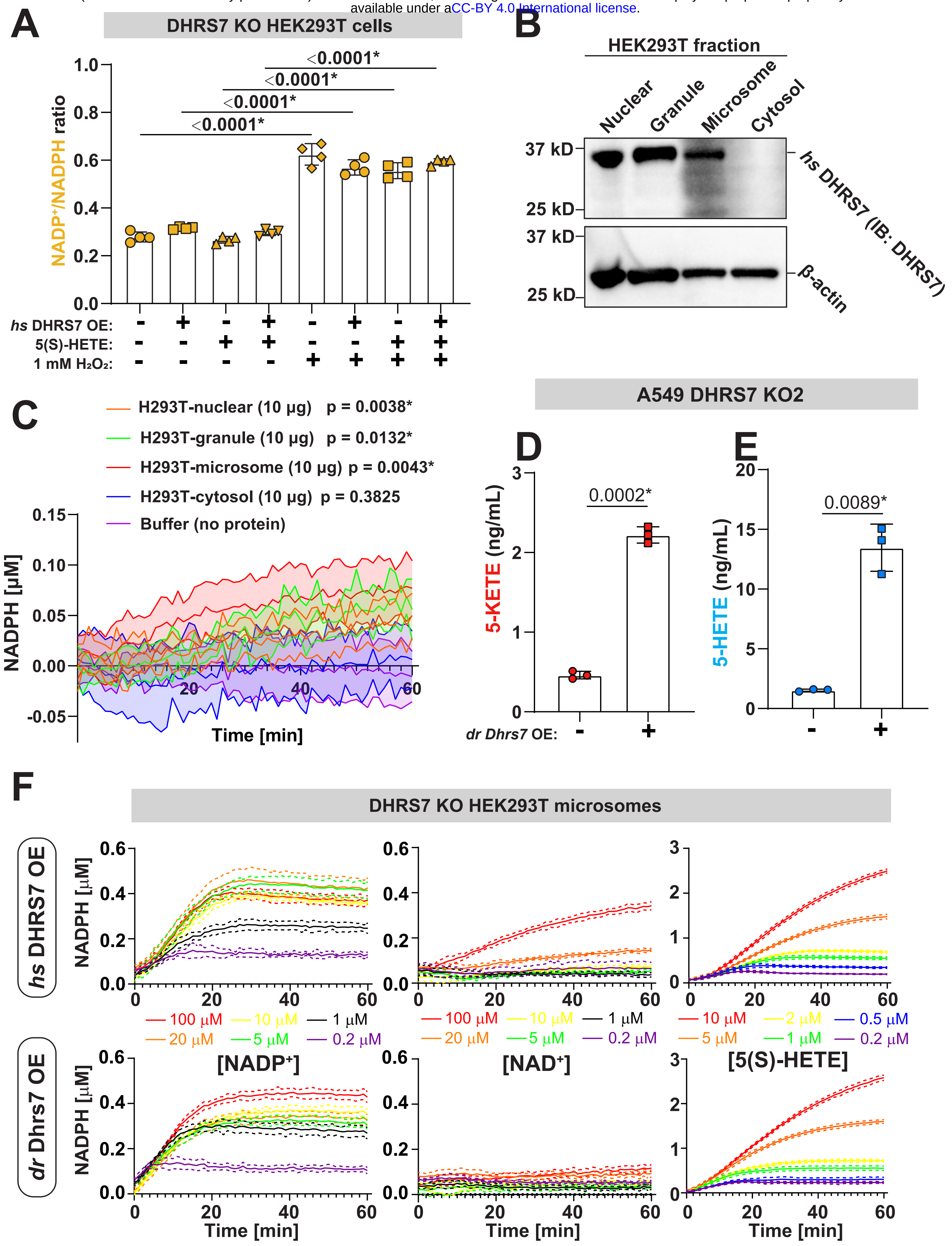

bioRxiv preprint doi: https://doi.org/10.1101/2025.02.05.636725; this version posted February 8, 2025. The copyright holder for this preprint (which was not certified by peer review) is the author/funder, who has granted bioRxiv a license to display the preprint in perpetuity. It is made available under aCC-BY 4.0 International license.

Figure S3. Extended characterization of DHRS7 as 5-HEDH, related to Figure 1-3.

(A) $NADP^+$/NADPH ratios in HEK293T DHRS7 KO cells ± *hs* DHRS7 OE, 1 μM 5(S)-HETE, or 1 mM H_2O_2 (20 min). P values, one-way ANOVA (uncorrected Fisher's LSD test, two-tailed). **(B)** Western blot assay for endogenous DHRS7 protein expression in nuclear, granule, microsome, cytosolic fractions of HEK293T cells. β-actin was used as loading control. **(C)** NADPH production assays probing for 5-HEDH activity in nuclear, granule, microsome and cytosolic fractions incubated with 1 μM 5(S)-HETE and 100 μM $NADP^+$. Statistical comparisons were performed at the 60 min time point. P values (microsomes), unpaired, nonparametric two tailed Mann-Whitney test. P values (other fractions), unpaired, parametric, two tailed, Welch's t-test. Shaded plot regions, SD. **(D-E)** LC-MS assays of 5-KETE or 5-HETE production in DHRS7-KO A549 cells ± *dr* Dhrs7-OE and lipid substrate incubation as indicated. Dhrs7 overexpression was induced with 1 μg/mL doxycycline. P values, unpaired, parametric, two tailed Welch's t test. Error bars, SD. **(F)** NADPH (or NADH) kinetics with 1.0975 pmol microsomal (top) *hs* DHRS7 or (bottom) *dr* Dhrs7 at the indicated, variable concentrations of (left) $NADP^+$, (middle) NAD^+, or (right) 5(S)-HETE, and (left-middle) 1 μM 5(S)-HETE, or (right) 100 μM $NADP^+$, respectively. The v_0 approximation of Figure 3H is based on the first 20 timepoints of the data in the left and right panels. Solid lines, mean values of data; dotted lines, SD. Asteriks, highlight significant changes as compared to respective control ($p<0.05$).

Figure S4

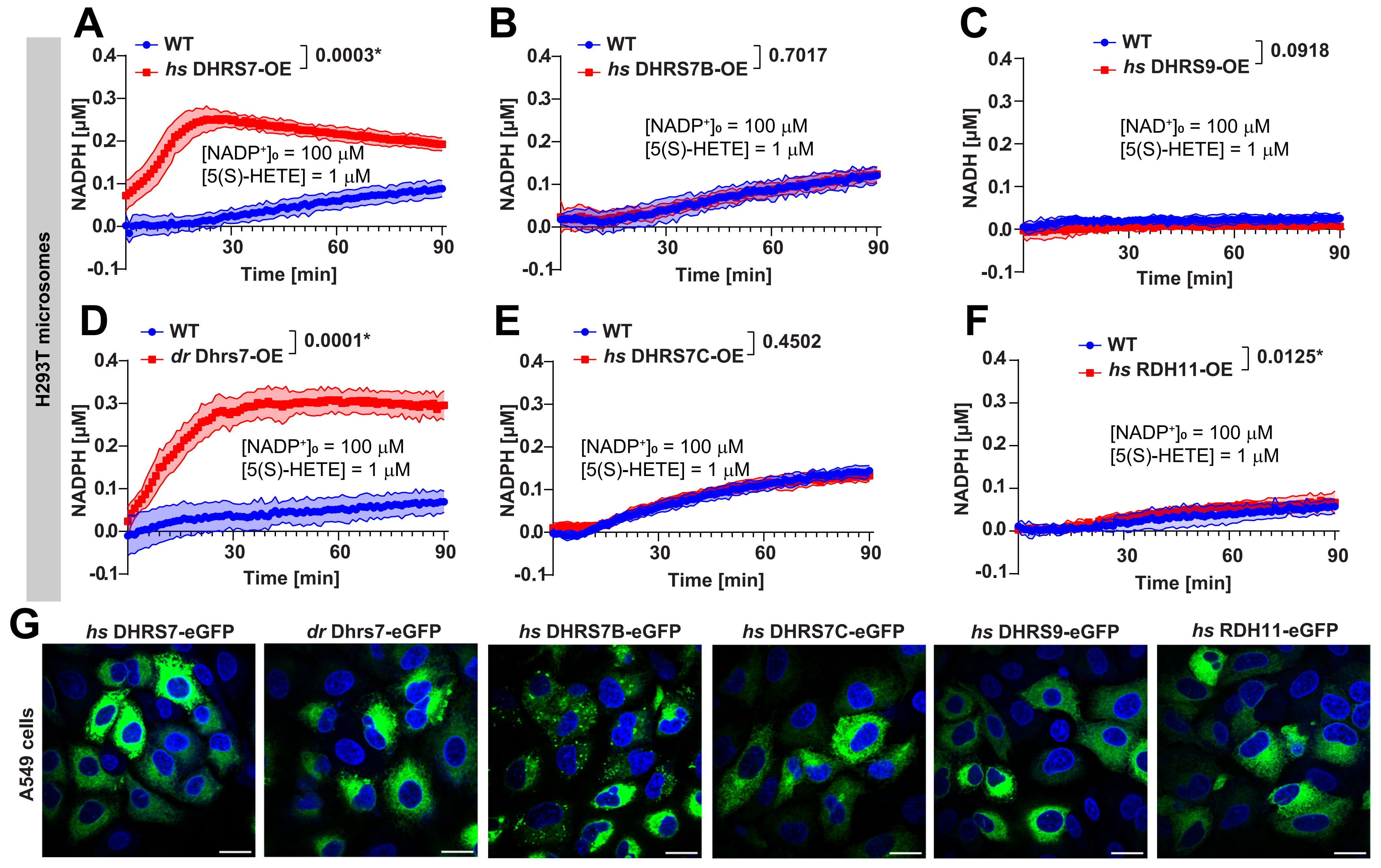
A
B
C
D
E
F
G
H293T microsomes
A549 cells
WT
hs DHRS7-OE
0.0003*
hs DHRS7B-OE
0.7017
hs DHRS9-OE
0.0918
dr Dhrs7-OE
0.0001*
hs DHRS7C-OE
0.4502
hs RDH11-OE
0.0125*
[NADP⁺]₀ = 100 µM
[NAD⁺]₀ = 100 µM
[5(S)-HETE] = 1 µM
NADPH [µM]
NADH [µM]
Time [min]
hs DHRS7-eGFP
dr Dhrs7-eGFP
hs DHRS7B-eGFP
hs DHRS7C-eGFP
hs DHRS9-eGFP
hs RDH11-eGFP

bioRxiv preprint doi: https://doi.org/10.1101/2025.02.05.636725; this version posted February 8, 2025. The copyright holder for this preprint (which was not certified by peer review) is the author/funder, who has granted bioRxiv a license to display the preprint in perpetuity. It is made available under aCC-BY 4.0 International license.

Figure S4. Extended characterization of DHRS7 as 5-HEDH, related to Figure 1-3.

NADPH or NADH production assays with *wt* HEK293T microsomes overexpressing **(A)** *hs* DHRS7, **(B)** *hs* DHRS7B, **(C)** *hs* DHRS9, **(D)** *dr* Dhrs7, **(E)** *hs* DHRS7C, or **(F)** *hs* RDH11. 10 µg microsomes were incubated with 1 µM 5(S)-HETE and 100 µM $NADP^+$, except for DHRS9 expressing microsomes, which were incubated with 1 µM 5(S)-HETE and 100 µM NAD^+. NADPH or NADH levels are background corrected (using DMSO as vehicle control without substrate). P values, unpaired, parametric, two-tailed, Welch's t test at the 20 min timepoint. Shaded plot regions, SD. **(G)** Representative images of the sub-cellular localization for the respective eGFP fusion proteins in A549 cells. Scale bar, 20 µm. Green, fusion protein. Blue, nucleus (DAPI). Asteriks, highlight significant changes as compared to respective control ($p<0.05$).

Figure S5

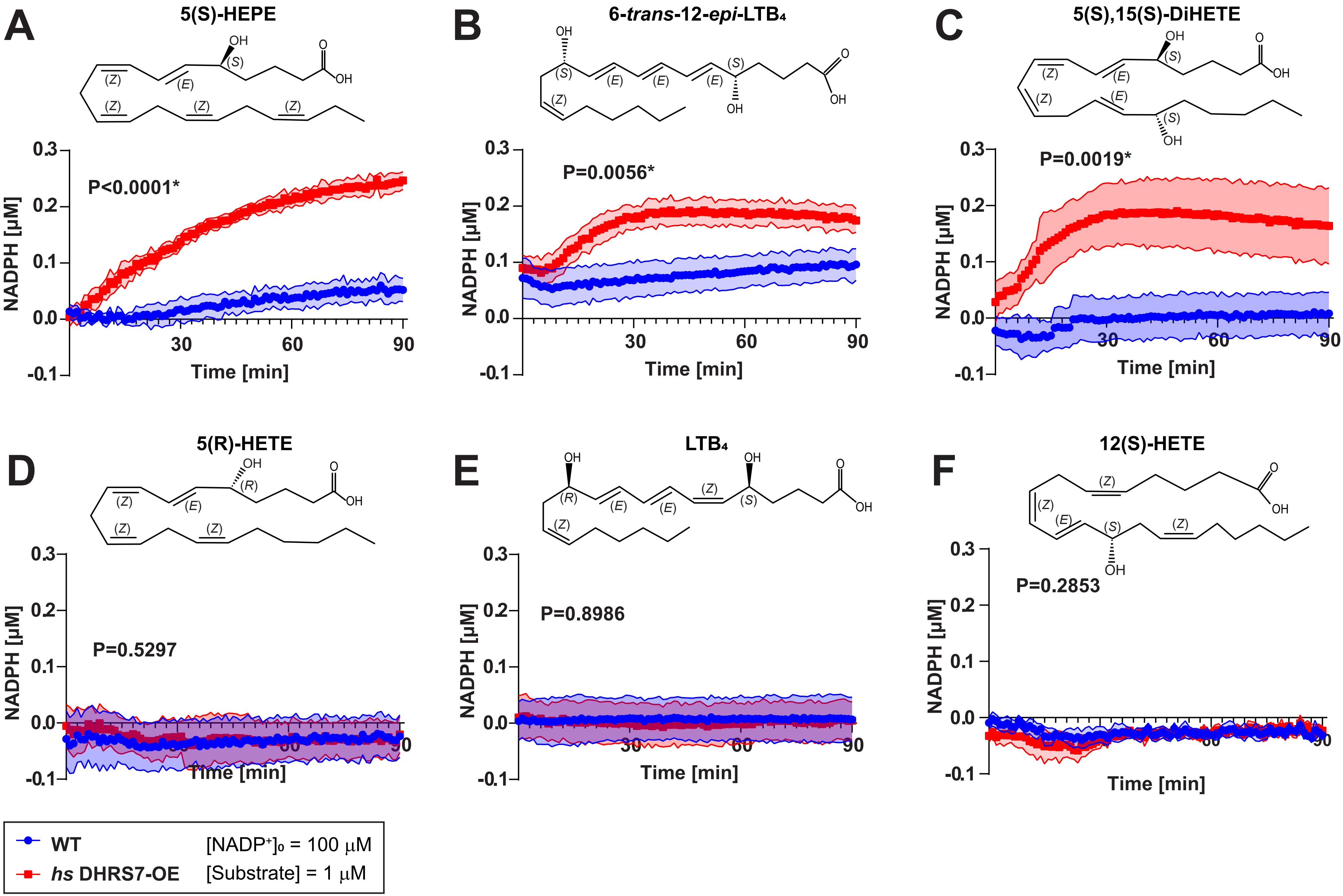

A
5(S)-HEPE
P<0.0001*
B
6-trans-12-epi-LTB4
P=0.0056*
C
5(S),15(S)-DiHETE
P=0.0019*
D
5(R)-HETE
P=0.5297
E
LTB4
P=0.8986
F
12(S)-HETE
P=0.2853
NADPH [µM]
Time [min]
WT
hs DHRS7-OE
[NADP+]0 = 100 µM
[Substrate] = 1 µM

bioRxiv preprint doi: https://doi.org/10.1101/2025.02.05.636725; this version posted February 8, 2025. The copyright holder for this preprint (which was not certified by peer review) is the author/funder, who has granted bioRxiv a license to display the preprint in perpetuity. It is made available under aCC-BY 4.0 International license.

Figure S5. Extended characterization of DHRS7 as 5-HEDH, related to Figure 1-3.

NADPH production assays were conducted with 100 µM $NADP^+$ and 1 µM **(A)** 5(S)-HEPE, **(B)** 6-*trans*-12-*epi* LTB_4, **(C)** 5(S),15(S)-DiHETE, **(D)** 5(R)-HETE, **(E)** LTB_4, and **(F)** 12(S)-HETE. NADPH levels are background corrected (using DMSO as vehicle control without substrate). P values, unpaired, parametric, two tailed, Welch's t test at the 20 min timepoint. Shaded plot regions, SD. Asterisks, highlight significant changes as compared to respective control ($p<0.05$).

Figure S6

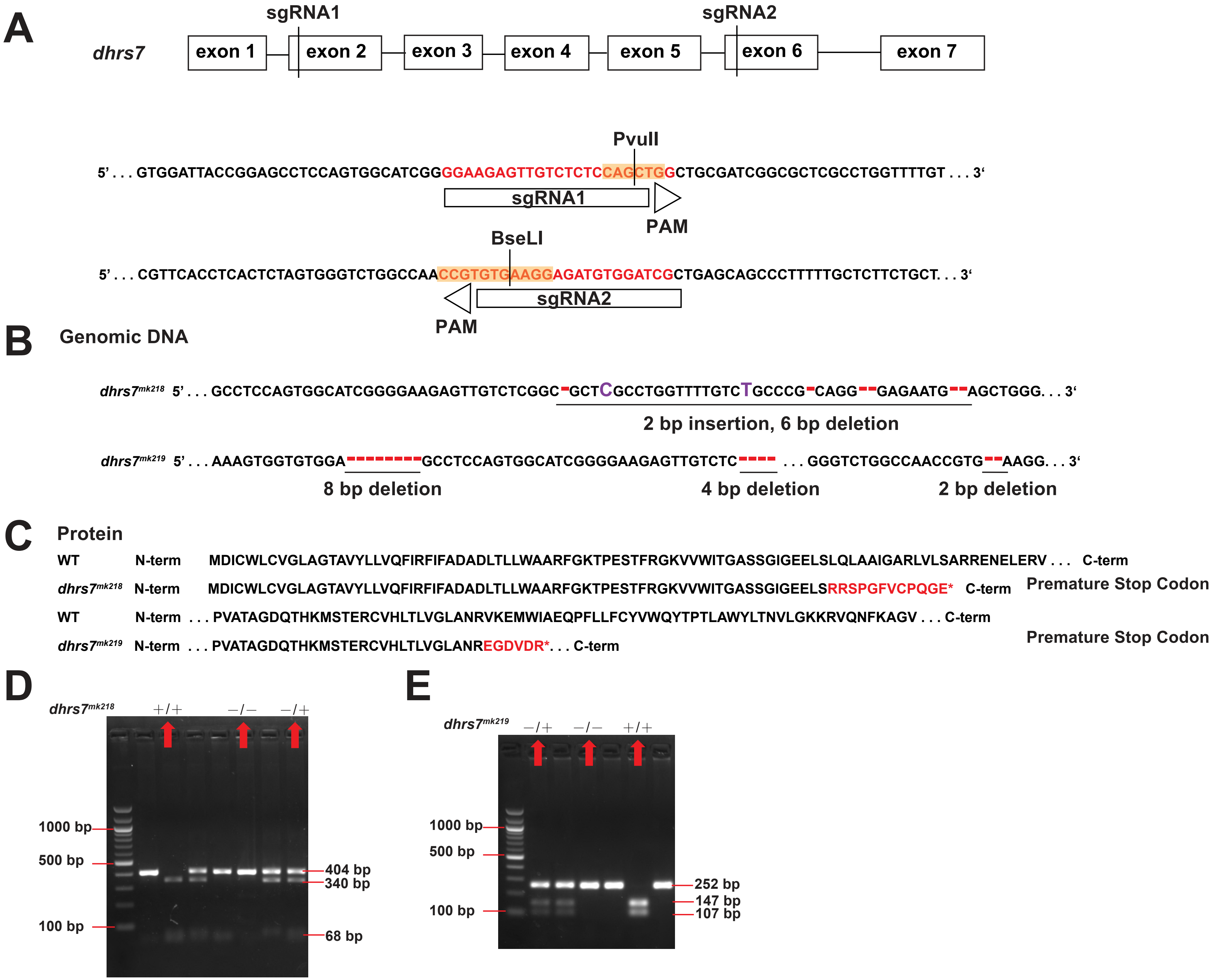
A
sgRNA1
sgRNA2
dhrs7
exon 1
exon 2
exon 3
exon 4
exon 5
exon 6
exon 7
PvuII
5'. . . GTGGATTACCGGAGCCTCCAGTGGCATCGGGGAAGAGTTGTCTCTCCAGCTGGCTGCGATCGGCGCTCGCCTGGTTTTGT . . . 3'
sgRNA1
PAM
BseLI
5' . . . CGTTCACCTCACTCTAGTGGGTCTGGCCAACCGTGTGAAGGAGATGTGGATCGCTGAGCAGCCCTTTTTGCTCTTCTGCT. . . 3'
sgRNA2
PAM
B
Genomic DNA
dhrs7mk218 5' . . . GCCTCCAGTGGCATCGGGGAAGAGTTGTCTCGGC-GCTCGCCTGGTTTTGTCTGCCCG-CAGG--GAGAATG--AGCTGGG. . . 3'
2 bp insertion, 6 bp deletion
dhrs7mk219 5' . . . AAAGTGGTGTGGA--------GCCTCCAGTGGCATCGGGGAAGAGTTGTCTC---- . . . GGGTCTGGCCAACCGTG--AAGG. . . 3'
8 bp deletion
4 bp deletion
2 bp deletion
C
Protein
WT N-term MDICWLCVGLAGTAVYLLVQFIRFIFADADLTLLWAARFGKTPESTFRGKVVWITGASSGIGEELSLQLAAIGARLVLSARRENELERV . . . C-term
dhrs7mk218 N-term MDICWLCVGLAGTAVYLLVQFIRFIFADADLTLLWAARFGKTPESTFRGKVVWITGASSGIGEELSRRSPGFVCPQGE* C-term
Premature Stop Codon
WT N-term . . . PVATAGDQTHKMSTERCVHLTLVGLANRVKEMWIAEQPFLLFCYVWQYTPTLAWYLTNVLGKKRVQNFKAGV . . . C-term
dhrs7mk219 N-term . . . PVATAGDQTHKMSTERCVHLTLVGLANREGDVDR*. . . C-term
Premature Stop Codon
D
dhrs7mk218
+/+
−/−
−/+
1000 bp
500 bp
100 bp
404 bp
340 bp
68 bp
E
dhrs7mk219
−/+
−/−
+/+
1000 bp
500 bp
100 bp
252 bp
147 bp
107 bp

bioRxiv preprint doi: https://doi.org/10.1101/2025.02.05.636725; this version posted February 8, 2025. The copyright holder for this preprint (which was not certified by peer review) is the author/funder, who has granted bioRxiv a license to display the preprint in perpetuity. It is made available under aCC-BY 4.0 International license.

Figure S6. Generation of *dhrs7* mutant zebrafish lines, related to Figure 4.

(A) Schematic diagram showing the design of single guide RNAs (sgRNAs) to generate *dhrs7* CRISPR zebrafish. For *dhrs7*$^{mk218/mk218}$, sgRNA1 was designed to target exon 2. Target disruption is predicted to occur at a PvuII restriction site (CAG^CTG) (highlighted by orange text) in the genomic DNA (deoxyribonucleic acid) sequence. For *dhrs7*$^{mk219/mk219}$, two different sgRNAs were used together. Specifically, sgRNA2 was designed to target exon 6. Target disruption is predicted to occur at a BseLI restriction site (CCGTGTG^AAGG) (highlighted by orange text) in the genomic DNA sequence. **(B)** *dhrs7* mutant alleles. The mutant *dhrs7*$^{mk218/mk218}$ allele was generated by sgRNA1 and has a 4-base pair (bp) deletion resulting in a frameshift and the destruction of PvuII restriction site. The mutant *dhrs7*$^{mk219/mk219}$ allele was generated by co-injection of sgRNA1 and sgRNA2 and has a 12 bp deletion leading to the destruction of a PvuII restriction site, as well as a 2 bp deletion and the destruction of a BseLI restriction site, resulting in a frame shift. Purple text, mutant sequence (insertion) that differs from the *wt* sequence. Red text, deletion sequence that differs from the *wt* sequence. **(C)** The mutant *dhrs7*^{mk218} and *dhrs7*^{mk219} alleles both have premature stop codons in the coding sequence. Red text, mutant *dhrs7*^{mk218} and *dhrs7*^{mk219} sequences that differs from the *wt* sequence. **(D-E)** Heterozygous *dhrs7*^{mk218} or *dhrs7*^{mk219} zebrafish were crossed, and genomic DNA isolated from the progeny was PCR-amplified (PvuII-digested for *dhrs7*$^{mk218/mk218}$, BseLI-digested for *dhrs7*$^{mk219/mk219}$), and genotyped by agarose gel electrophoresis. As for *dhrs7*$^{mk218/mk218}$ (left panel), the PCR products from *dhrs7-1*$^{wt/wt}$ fish are cleaved into two smaller products by PvuII (340 bp and 68 bp products). PCR products from homozygous *dhrs7-1*$^{mk218/mk218}$ mutant fish are not cleaved by PvuII (404 bp product only). PCR products from heterozygote *dhrs7-1*$^{wt/mk218}$ fish are identified by the presence of a cleaved *wt* allele and non-cleaved mutant allele (404, 340 and 68 bp cleavage products). As for *dhrs7*$^{mk219/mk219}$ (right panel), the PCR products from *dhrs7-1*$^{wt/wt}$ fish are cleaved into two smaller products by BseLI (147 bp and 107 bp cleavage products). PCR products from homozygous mutant *dhrs7*$^{mk219/mk219}$ fish are not cleaved by BseLI (252 bp product only). PCR products from heterozygotes of *dhrs*$^{wt/mk219}$ were identified by the presence of a cleaved *wt* allele and non-cleaved mutant allele (252, 147 and 107 bp cleavage products).

Figure S7

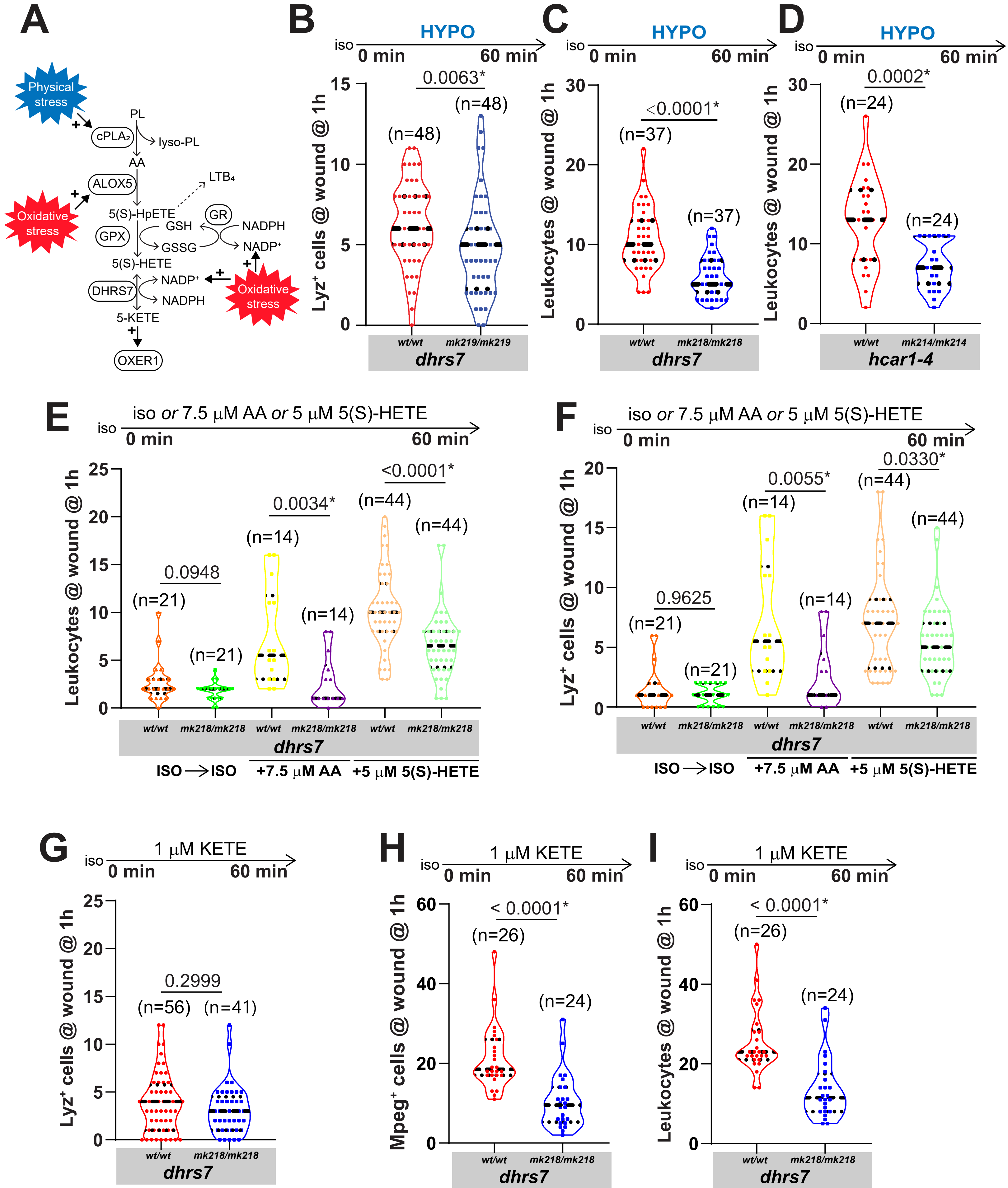
A
Physical stress
Oxidative stress
PL
cPLA₂
lyso-PL
AA
ALOX5
LTB₄
5(S)-HpETE
GR
GSH
NADPH
GPX
GSSG
NADP⁺
5(S)-HETE
NADP⁺
DHRS7
NADPH
Oxidative stress
5-KETE
OXER1
B
HYPO
iso
0 min
60 min
Lyz⁺ cells @ wound @ 1h
0.0063*
(n=48)
(n=48)
wt/wt
mk219/mk219
dhrs7
C
HYPO
iso
0 min
60 min
Leukocytes @ wound @ 1h
<0.0001*
(n=37)
(n=37)
wt/wt
mk218/mk218
dhrs7
D
HYPO
iso
0 min
60 min
Leukocytes @ wound @ 1h
0.0002*
(n=24)
(n=24)
wt/wt
mk214/mk214
hcar1-4
E
iso or 7.5 μM AA or 5 μM 5(S)-HETE
iso
0 min
60 min
Leukocytes @ wound @ 1h
0.0948
0.0034*
<0.0001*
(n=21)
(n=21)
(n=14)
(n=14)
(n=44)
(n=44)
wt/wt
mk218/mk218
wt/wt
mk218/mk218
wt/wt
mk218/mk218
dhrs7
ISO → ISO
+7.5 μM AA
+5 μM 5(S)-HETE
F
iso or 7.5 μM AA or 5 μM 5(S)-HETE
iso
0 min
60 min
Lyz⁺ cells @ wound @ 1h
0.9625
0.0055*
0.0330*
(n=21)
(n=21)
(n=14)
(n=14)
(n=44)
(n=44)
wt/wt
mk218/mk218
wt/wt
mk218/mk218
wt/wt
mk218/mk218
dhrs7
ISO → ISO
+7.5 μM AA
+5 μM 5(S)-HETE
G
1 μM KETE
iso
0 min
60 min
Lyz⁺ cells @ wound @ 1h
0.2999
(n=56)
(n=41)
wt/wt
mk218/mk218
dhrs7
H
1 μM KETE
iso
0 min
60 min
Mpeg⁺ cells @ wound @ 1h
< 0.0001*
(n=26)
(n=24)
wt/wt
mk218/mk218
dhrs7
I
1 μM KETE
iso
0 min
60 min
Leukocytes @ wound @ 1h
< 0.0001*
(n=26)
(n=24)
wt/wt
mk218/mk218
dhrs7

bioRxiv preprint doi: https://doi.org/10.1101/2025.02.05.636725; this version posted February 8, 2025. The copyright holder for this preprint (which was not certified by peer review) is the author/funder, who has granted bioRxiv a license to display the preprint in perpetuity. It is made available under aCC-BY 4.0 International license.

Figure S7. Characterization of Dhrs7 function in live zebrafish, related to Figure 4.

(A) Schematic diagram depicting the oxoeicosanoid pathway. $cPla_2$ is activated by physical stress (specifically, osmotic nuclear deformation) to release arachidonic acid (AA) from nuclear membrane phospholipids (PL). ALOX5 (5-lipoxygenase) metabolizes AA into 5-hydroperoxy-eicosatetraenoic acid (5(S)-HpETE). The catalytic site of ALOX5 is primed by free lipid peroxides (i.e., oxidative stress). 5(S)-HpETE may be either used for LTB_4 production or reduced by a glutathione peroxidase (GPX) into 5-hydroxyeicosatetraenoic acid (5(S)-HETE). The respective oxidized glutathione (GSSG) is converted back to reduced glutathione (GSH) by glutathione reductase (GR) under consumption of NADPH and generation of $NADP^+$. Under oxidative (=high $NADP^+$) and physical stress (=high 5(S)-HETE), 5-KETE is generated from 5(S)-HETE. Under unstressed conditions, the reverse reaction is preferred. 5-KETE exerts its physiological effects through the OXER1 receptor (or its zebrafish ortholog Hcar1-4). **(B)** Quantification of Lyz^+ neutrophils at the wound margins of *dhrs7*$^{mk219/mk219}$ or *wt* control siblings at 1 hour. **(C-D)** Quantification of “generic” leukocytes (identified by transmitted light contrast and motility) at the wound margins of **(C)** *dhrs7*$^{mk218/mk218}$ **(D)** *hcar1-4*$^{mk214/mk214}$ zebrafish compared to their respective control siblings (same experiment as in Figure 4C-D). **(E)** Quantification of Lyz^+ neutrophils or **(F)** “generic” leukocytes at wound margin of *dhrs7*$^{mk218/mk218}$ mutant and sibling control larvae treated with the indicated lipids and isotonic bathing solution (Tg(*lyz*:pm2-mKate2) background). Quantification of **(G)** Lyz+ neutrophils, **(H)** Mpeg+ macrophages, or **(I)** “generic” leukocytes at the wound margins of *dhrs7*$^{mk218/mk218}$ mutant or *wt* sibling larvae after 1 hour of incubation with 1 µM 5-KETE in isotonic bathing solution (Tg(*lyz*:pm2-mk2) or Tg(*mpeg1*:eGFP) backgrounds). Dashed violin plot lines, first quartiles (top line), medians (middle line), third quartiles (bottom line). Parentheses, total number of wounded larvae. P values, unpaired, nonparametric, two-tailed Mann-Whitney U test. Note, (H-I) refer to the same experiment. Asterisks, highlight significant changes as compared to respective control (p<0.05).

bioRxiv preprint doi: https://doi.org/10.1101/2025.02.05.636725; this version posted February 8, 2025. The copyright holder for this preprint (which was not certified by peer review) is the author/funder, who has granted bioRxiv a license to display the preprint in perpetuity. It is made available under aCC-BY 4.0 International license.

Movie Legends

Video S1. Live imaging of neutrophil recruitment to the wound margin of *dhrs7*$^{wt/wt}$ and *dhrs7* $^{mk218/mk218}$ larvae (tg(*lyz*:pm2-mk2) background), related to Figure 4C.

Representative time-lapse movie showing neutrophil recruitment to the wound margin. After 5-minute bathing in isotonic buffer, the bathing solution was switched to hypotonic buffer to trigger the wound response.

Video S2. Live imaging of neutrophil recruitment to the wound margin of *hcar1-4*$^{wt/wt}$ and *hcar1-4* $^{mk214/mk214}$ larvae (tg(*lyz*:pm2-mk2) background), related to Figure 4D.

Representative time-lapse movie showing neutrophil recruitment to the wound margin. After 5-minute bathing in isotonic buffer, the bathing solution was switched to hypotonic buffer to trigger the wound response.

Video S3. Live imaging of macrophage recruitment to wound margin in *dhrs7*$^{wt/wt}$ and *dhrs7* $^{mk218/mk218}$ larvae (tg(*mpeg1*:eGFP) background), related to Figure 4E.

Representative time-lapse movie showing macrophage recruitment to the wound margin. After 5-minute bathing in isotonic buffer, the bathing solution was switched to hypotonic buffer to trigger the wound response.

Video S4. Live imaging of macrophage recruitment to wound margin in *hcar1-4*$^{wt/wt}$ and *hcar1-4* $^{mk214/mk214}$ larvae (tg(*mpeg1*:eGFP) background), related to Figure 4F.

Representative time-lapse movie showing macrophage recruitment to the wound margin. After 5-minute bathing in isotonic buffer, the bathing solution was switched to hypotonic buffer to trigger the wound response.
